# Spray-Drying Synthesis of LiFeBO_3_/C Hollow Spheres With Improved Electrochemical and Storage Performances for Li-Ion Batteries

**DOI:** 10.3389/fchem.2019.00379

**Published:** 2019-05-28

**Authors:** Yulei Sui, Wei Chen, Shibao Tang, Ling Wu, Binjue Wang, Huacheng Li, Wei Li, Shengkui Zhong

**Affiliations:** ^1^School of Iron and Steel, Soochow University, Suzhou, China; ^2^Citic Dameng Mining Industries Limited, Chongzuo, China; ^3^Guangxi Key Laboratory of Electrochemical and Magnetochemical Functional Materials, Guilin University of Technology, Guili, China

**Keywords:** Li-ion batteries, LiFeBO_3_, hollow sphere, cathode materials, spray drying

## Abstract

LiFeBO_3_/C cathode material with hollow sphere architecture is successfully synthesized by a spray-drying method. SEM and TEM results demonstrate that the micro-sized LiFeBO_3_/C hollow spheres consist of LiFeBO_3_@C particles and the average size of LiFeBO_3_@C particles is around 50–100 nm. The thickness of the amorphous carbon layer which is coated on the surface of LiFeBO_3_ nanoparticles is about 2.5 nm. LiFeBO_3_@C particles are connected by carbon layers and formed conductive network in the LiFeBO_3_/C hollow spheres, leading to improved electrical conductivity. Meanwhile, the hollow structure boosts the Li^+^ diffusion and the carbon layers of LiFeBO_3_@C particles protect LiFeBO_3_ from moisture corrosion. Consequently, synthesized LiFeBO_3_/C sample exhibits good electrochemical properties and storage performance.

## Introduction

With the development of electric vehicles in the twenty-first century, Li-ion batteries (LIBs) play an increasingly important role in modern society (Nayak et al., 2018; Wu et al., 2019) and various cathode materials are developed (Ma et al., 2015; Zheng et al., 2018a,b; Yang et al., 2019). Recently, borate-based materials (LiMBO_3_, M = Fe, Mn, and Co) have received wide attention in the field of LIBs (Lin et al., 2012; Tao et al., 2013). Compared with the commonly used phosphate-based materials (LiMPO_4_, M = Fe, Mn, and Co), borate groups (LiMBO_3_, M = Fe, Mn, and Co) with a low specific weight show higher capacities. For instance, the theoretical capacity of LiMnBO_3_ is 222 mAh g^−1^, whereas the phosphate counterpart, LiMnPO_4_, is limited to 170 mAh g^−1^ (Moskon et al., 2016; Zhang et al., 2017). Moreover, the volume changes of borate-based materials during charging-discharging process are less than those of phosphate-based materials so that they usually show good structural stability (Loftager et al., 2017). Among various borate-based cathode materials, LiFeBO_3_ with the theoretical capacity of 220 mAh g^−1^ presents moderate working voltage, relatively high intrinsic conductivity, and good structural stability (Michalski et al., 2017). Therefore, LiFeBO_3_ is considered as a potential cathode material for LIBs. However, there is only one-dimensional (1D) pathway for Li^+^ diffusing in the LiFeBO_3_ crystal, which leading to low electronic and ionic conductivity (Dong et al., 2008; Kalantarian et al., 2013). Furthermore, LiFeBO_3_ is highly sensitive to moisture so that surface poisoning by H_2_O molecules of air would seriously disrupt its electrochemical properties (Gu et al., 2017). These problems make it challenging to achieve LiFeBO_3_-based materials with high reversible specific capacity and good storage performance. In order to improve the electrochemical performance of LiFeBO_3_-based materials, various strategies have been introduced, such as metal-ions doping, conductive carbon coating, and nano-architecturing (Aravindan and Umadevi, 2012; Afyon et al., 2013; Le Roux et al., 2015; Sin et al., 2015). Among these methods, carbon coating is a promising and effective way to improve the properties of LiFeBO_3_, which attributes to the improved conductivity and the prevention of moisture corrosion. For example, Li et al. reported that the carbon coated LiFeBO_3_ nanoparticles exhibit the improved first discharge capacities of 190.4 and 106.6 mAh g^−1^ at 0.1 and 1°C rate, respectively (Li et al., 2015). The LiFeBO_3_/C prepared by Zhang et al. also indicated carbon coating is an effective way to improve the properties of LiFeBO_3_. However, the rate capability and storage performance of LiFeBO_3_ are still not satisfied (Zhang et al., 2014). Therefore, achieving homogeneous carbon coated LiFeBO_3_ cathode materials with high electrochemical properties and good storage performance is still a challenge right now.

Herein, LiFeBO_3_/C with hollow porous sphere architecture is successfully synthesized by spray-drying method at the temperature as low as 450°C (Li et al., 2015). Amorphous carbon layer which was produced by polyethylene glycol 6,000 (PEG-6000) decomposition is coated on the surface of LiFeBO_3_ nanoparticles. The LiFeBO_3_@C particles are connected together and formed LiFeBO_3_/C hollow porous spheres. The obtained LiFeBO_3_/C sample with distinctive architecture shows the improved electrochemical and storage performances as cathode for LIBs.

## Experimental

### Synthesis of LiFeBO_3_/C and LiFeBO_3_

Firstly, LiNO_3_, 1.38g LiNO_3_, 8.08g Fe(NO_3_)_3_·9H_2_O, and 1.24g H_3_BO_3_ were weighed and dissolved gradually in deionized water under stirring, and the mixed solution was named solution A. Meanwhile, 1.22 g PEG-6000 was dropped and dissolved in deionized water under stirring to form solution B. Then the solution B was added into the solution A and stirred carefully. A homogeneous sol was obtained when the mixed solution has been stirred at 80°C for 1 h. The precursor of LiFeBO_3_/C was synthesized using the obtained sol as raw material via a spray drying process. The inlet and outlet air temperatures are 200 and 100°C during the spray drying process with the air pressure of 0.25 MPa. The as-obtained precursor was calcined at 350°C (3 h), followed by crystallized at 450°C for 10 h in an argon atmosphere, and the final LiFeBO_3_/C was obtained. For contrast, the LiFeBO_3_ sample was also prepared by the similar synthetic process but without PEG-6000. All the chemical regents employed in this work were of analytic grade.

### Characterization

The structure of LiFeBO_3_/C and LiFeBO_3_ samples was investigated by XRD (Rigaku/Ultima-IV) and Raman micro-spectroscopy. And the range of XRD and Raman spectroscopic analysis is 2θ = 10 ~ 80° and 600–2,000 cm^−1^, respectively. Morphology and microstructure of LiFeBO_3_/C and LiFeBO_3_ samples were investigated by SEM (JSM/6380LV) and TEM (TecnaiG220). C-S analysis (Eltar) was used to determine the carbon content of samples.

### Electrochemical Measurements

The electrochemical measurements were performed in coin-type cells (CR2025) with lithium metal as the negative electrode. The positive electrodes were prepared by mixing as-prepared LiFeBO_3_/C or LiFeBO_3_ powder, acetylene black (99.9%, Sinopharm Chemial Reagent Co., Ltd.) and polyvinylidene fluoride (PVDF, 99.9%, Sinopharm Chemial Reagent Co., Ltd. the weight ratio is 80:10:10) in N-methylpyrolline onto an Al foil and dried at 120°C for 4 h in vacuum oven. Then the coin-type cells were assembled in a glove box filled with high purity argon. 1M LiPF_6_ solution in a mixture of ethylene carbonate and dimethyl carbonate with 1:1 volumetric ratio was used as electrolyte (Sinopharm Chemial Reagent Co., Ltd.). The cells were tested in the voltage range of 1.5–4.5 V under various charge/discharge current densities from 5 to 100 mA g^−1^ at room temperature. The electrochemical impedance spectroscopy (EIS) and the cyclic voltammetry (CV) was both measured by a CHI 660D workstation.

## Results and Discussion

**Figure 1:**
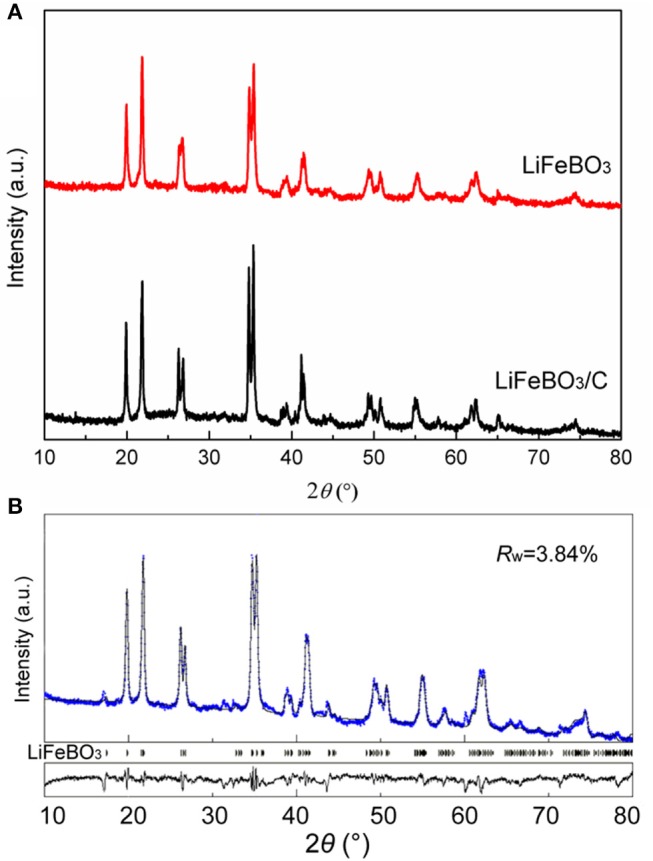
**(A)** XRD patterns of the as-prepared LiFeBO_3_/C and LiFeBO_3_; **(B)** Rietveld refinement XRD pattern of the as-prepared LiFeBO_3_/C.

XRD patterns of the synthesized LiFeBO_3_/C and LiFeBO_3_ samples are shown in Figure 1A. Obviously, the LiFeBO_3_ with monoclinic structure is well-crystallized. XRD patterns of two samples are similar, indicating the combination of LiFeBO_3_ and carbon has little influence on the crystal structure. Although the amount of carbon in the LiFeBO_3_/C composites is 4.9 wt. % (proved by C-S analysis), the diffraction peak of carbon is not detected from the XRD patterns, implying the carbon in the LiFeBO_3_/C composite is amorphous.

In order to further clarify the crystal structure and lattice parameters of the synthesized LiFeBO_3_/C, XRD data was refined by Rietveld method (Rietveld, 1967) using Maud software (Lutterotti, 2011), and the Rietveld refinement XRD pattern and atoms positions and occupancy for Li and Fe atoms in LiFeBO_3_/C investigated. As can be seen from Figure 1B and Table 1, the sharp peaks of the sample can be identified as monoclinic LiFeBO_3_ with space group of C2/c and the structure is crystallized well. Meanwhile, the observed and calculated patterns match well, and the reliability factors are good. In accordance with the refinement results, the lattice parameters of LiFeBO_3_ are *a* = 5.1662 Å, *b* = 8.9141 Å, *c* = 10.1700 Å, β = 91.25°, agreeing well with the reported literature (Zhang et al., 2014).

**Table 1:** Results of structural analysis obtained from X-ray Rietveld refinement of LiFeBO_3_/C.

**Atom**	**Site**	***x***	***Y***	***z***	**Occupancy**
Li1	8f	0.6175	0.5429	0.1539	0.48
Li2	8f	0.6573	0.4782	0.0605	0.52
Fe1	8f	0.1372	0.3332	0.1392	0.72
Fe2	8f	0.1835	0.3435	0.0915	0.28
B1	8f	0.1592	0.6431	0.1043	1
O1	8f	0.3877	0.1737	0.0891	1
O2	8f	0.7673	0.3031	0.1497	1
O3	8f	0.3229	0.5376	0.1216	1
Lattice parameters		a (Å)	b (Å)	c (Å)	β (°)
Sample		5.1662	8.9141	10.1700	91.25

**Figure 2:**
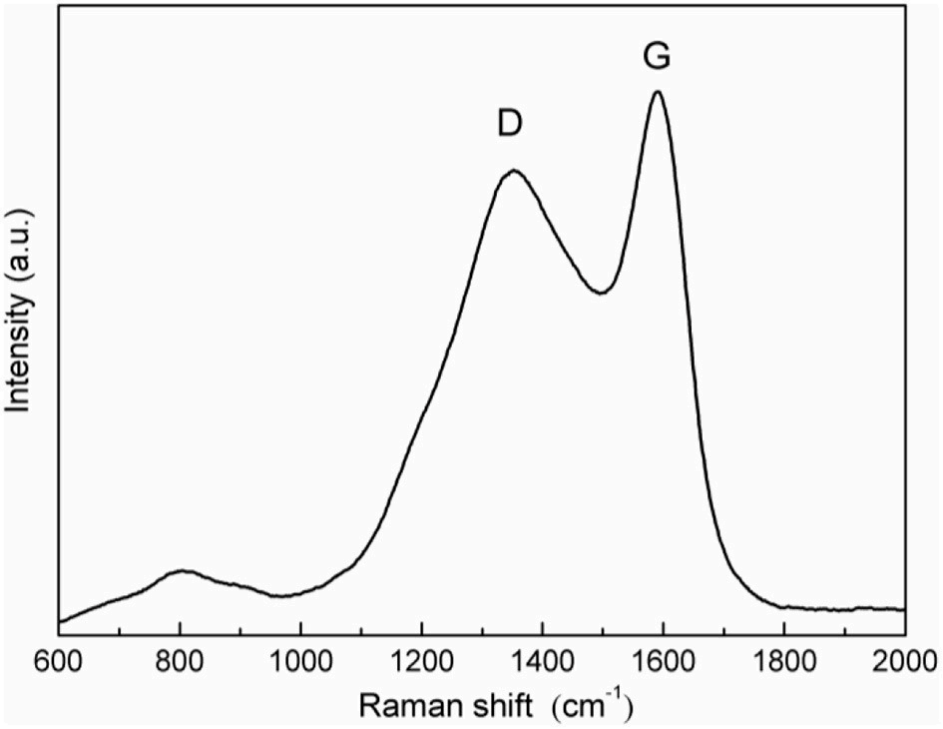
Raman spectra of the as-prepared LiFeBO_3_/C.

Figure 2 represents the Raman spectroscopy of the as-prepared LiFeBO_3_/C sample. As can be seen from Figure 2, the broad peak observed at 1,354 cm^−1^ correspond to the D (disordered) band of carbon while the broad peak located at 1,590 cm^−1^ agreeing well with G (graphitized) band of sp^2^ type carbon (Liu et al., 2015; Zhu et al., 2017). The strong D-peak further reveals that the carbon is amorphous, which is in agreement with the XRD result.

**Figure 3:**
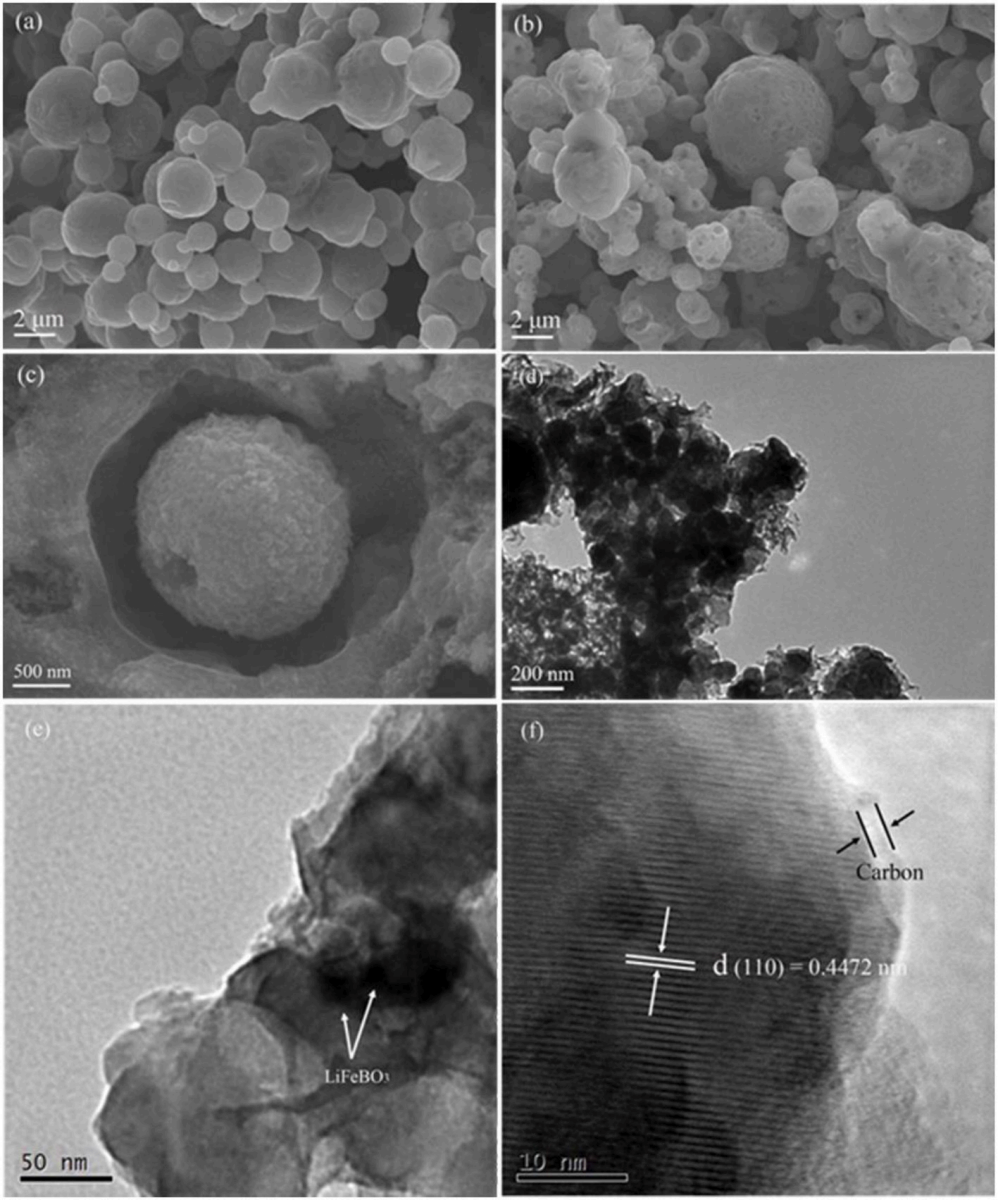
SEM images of the as-prepared LiFeBO_3_ **(a)** and LiFeBO_3_/C **(b,c)**; TEM images of the as-prepared LiFeBO_3_/C **(d–f)**.

SEM and TEM were employed to analyze morphology and micro-structure of the synthesized LiFeBO_3_ and LiFeBO_3_/C. As shown in Figure 3a, the LiFeBO_3_ sample displays a spherical shape with a size distribution in the range of 1–4 μm and the surface is compact. While, the LiFeBO_3_/C sample exhibits hollow spherical shape with a rough and porous surface layer (Figures 3b,c). The comparison of the darker outer shell and the lighter central regions indicates that the LiFeBO_3_/C spheres present porous and hollow internal structure (Figure 3d), which is consistent with the SEM results. As shown in Figure 3c, it is obvious that the spherical LiFeBO_3_/C are actually consist of numerous primary nanoparticles. Figure 3e shows that nano-sized primary LiFeBO_3_ particles are well wrapped by thick carbon layer. This carbon-coated layer is benefit to prevent LiFeBO_3_ from moisture corrosion. As we can see from Figure 3f, the lattice fringe with an interior planar distance of 0.4472 nm is correspond well to (1 1 0) crystal planes of LiFeBO_3_. The coating layer with the thickness of 2.5 nm could be assigned to the amorphous carbon, so that LiFeBO_3_@C shows a core-shell structure. In addition, the carbon-coated layer and conductive carbon network can improve the conductivity of LiFeBO_3_-based cathode material, which is also benefit to the electrochemical performances.

**Figure 4:**
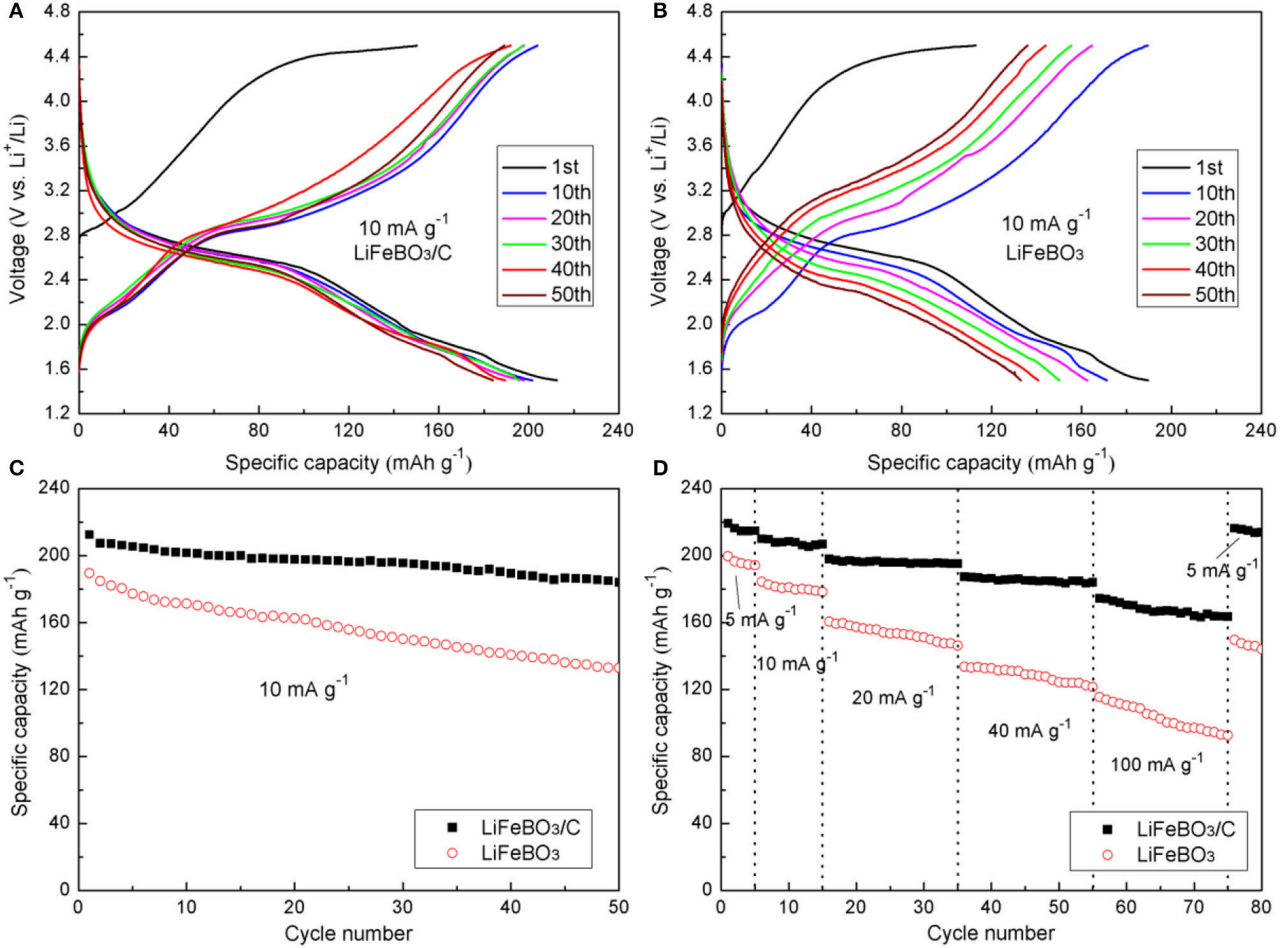
Charge-discharge curves of the LiFeBO_3_/C **(A)** and LiFeBO_3_ **(B)** at the current density of 10 mA g^−1^; **(C)** Cycle performance of LiFeBO_3_/C and LiFeBO_3_ at the current density of 10 mA g^−1^; **(D)** Rate capability of LiFeBO_3_/C and LiFeBO_3_ at various current densities.

In the potential (*vs*. Li/Li^+^) range of 1.5–4.5 V, the cells were charged/discharged at 10 mA g^−1^, and the curves of the as-synthesized LiFeBO_3_/C and LiFeBO_3_ samples are exhibited in Figures 4A,B, respectively. It is noted that the initial charge curves of both samples show obviously higher voltage plateau than the subsequent cycles, which mainly due to the high polarization before cycling. This phenomenon is very similar to cases of LiMnBO_3_ and LiCoBO_3_ (Afyon et al., 2014; Tang et al., 2015). The charge plateau (~3.2 V) and the discharge plateau (~2.8 V) in the voltage profiles correspond to the plateaus of LiFeBO_3_. There is a reduced thermodynamic potential for the degraded LiFeBO_3_ phase so that a short discharge plateau ~1.8 V appeared in the discharge profiles (Bo et al., 2012).

The cycling performances of the as-prepared LiFeBO_3_/C and LiFeBO_3_ samples at 10 mA g^−1^ are shown in Figure 4C. The initial discharge specific capacities of LiFeBO_3_/C and LiFeBO_3_ are 212.4 and 189.5 mAh g^−1^, respectively. The specific capacity of LiFeBO_3_/C is as high as 184.1 mAh g^−1^ while the specific capacity of LiFeBO_3_ is only 132.9 mAh g^−1^ after 50 cycles, indicating the LiFeBO_3_/C sample has a better cyclic stability. Figure 4D displays the rate capability of the as-prepared LiFeBO_3_/C and LiFeBO_3_ samples under different current densities. The initial capacity of the as-prepared LiFeBO_3_/C is 219.2 mAh g^−1^ at 5 mA g^−1^, which is approximately 98.7% of the theoretical capacity. When the current density is increased up to 40 and 100 mA g^−1^, the LiFeBO_3_/C sample holds a stable reversible capacity of 187.3 and 175.8 mAh g^−1^, respectively. However, the discharge capacity of LiFeBO_3_ at 40 and 100 mA g^−1^ are only 144.5 and 115.5 mAh g^−1^, respectively. If the current density returns back to 5 mA g^−1^ after 75 cycles test, and 98.6% of the initial discharge specific capacity (216.3 mAh g^−1^) can be recovered for LiFeBO_3_/C but only 75.1% was recovered for LiFeBO_3_ electrode (149.6 mAh g^−1^), implying the LiFeBO_3_/C sample has a better capacity recovery ability than LiFeBO_3_. The improved electrochemical performances can be attributed to the special hollow sphere structure and network of amorphous carbon layer. The conductivity is improved by the network of amorphous carbon layer and the volume expansion during the charge and discharge progresses is againsted due to the special hollow sphere architecture provides a flexible structure, so that the electrochemical performance of the synthesized cathode is improved (Li et al., 2013).

**Figure 5:**
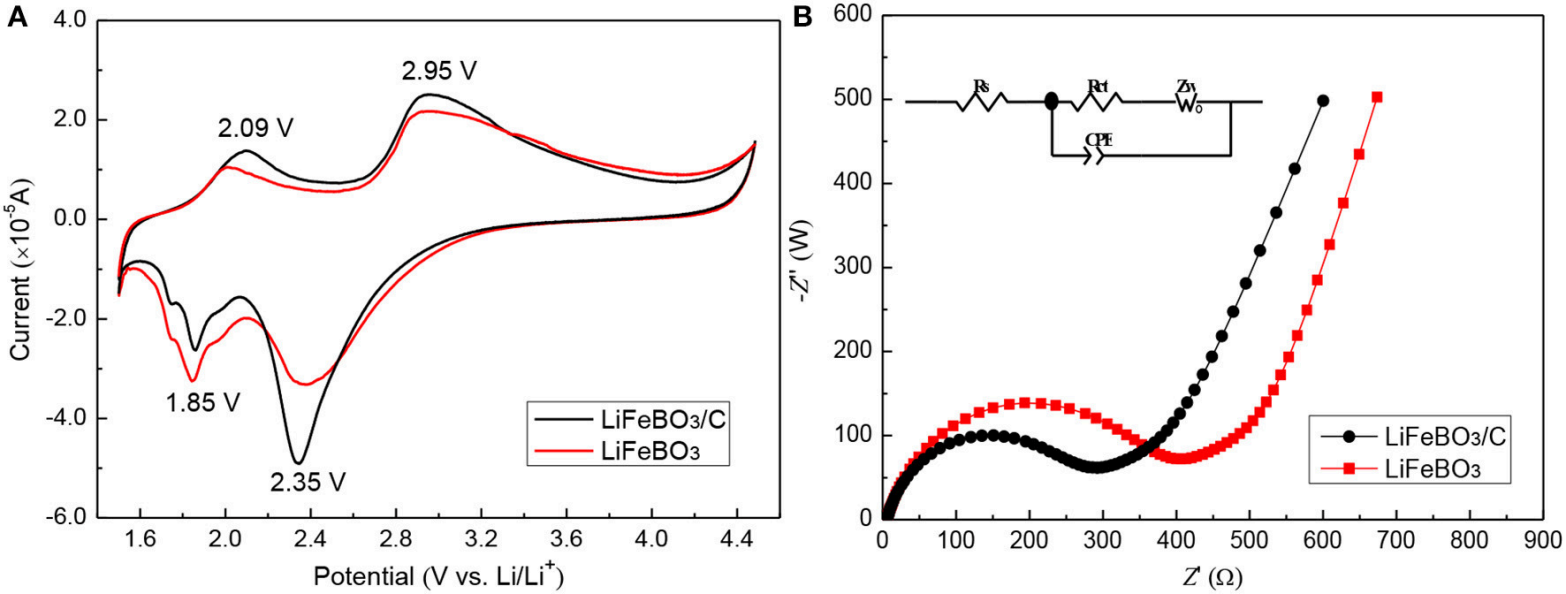
**(A)** Cyclic voltammetry profiles of LiFeBO_3_/C and LiFeBO_3_ at the scan rate of 0.1 mV s^−1^; **(B)** Electrochemical impedance spectra of as-prepared LiFeBO_3_/C and LiFeBO_3_.

Figure 5A shows the cyclic voltammetry (CV) of the as-prepared LiFeBO_3_/C and LiFeBO_3_ samples. The oxidation/reduction peaks located at ~2.95/2.35 V correspond to the phase transition between LiFeBO_3_ and FeBO_3_. It is clearly observed that LiFeBO_3_/C sample shows sharper peaks and larger areas than LiFeBO_3_, implying the synthesized LiFeBO_3_/C has a better reversibility. In addition, a couple of oxidation/reduction peaks are located at ~2.09/1.85 V, and these patterns can be ascribed to the degradation of LiFeBO_3_ (Afyon et al., 2014).

In order to analyze the conductivity of the as-prepared LiFeBO_3_/C and LiFeBO_3_ samples, the EIS tests (Figure 5B) are measured in our study. Then the EIS data is fitted with Z-view software using an equivalent circuit, and the related parameters are listed in Table 2. *R*_s_, *Z*_w_, and *R*_ct_ in the equivalent circuit (insert Figure 5B) represent the resistance of the electrolyte, Warburg impedance and charge transfer resistance, respectively. The *R*_ct_ of LiFeBO_3_/C (238.6 Ω) is lower than that of LiFeBO_3_ (354.1 Ω), implying the charge transfer speed during the charge and discharge processes of electrode is significantly improved. The exchange current density (*j*_0_) of synthesized LiFeBO_3_/C and LiFeBO_3_ is calculated by the following equation, respectively.

$$\begin{array}{l} j_{0}=\frac{RT}{nFR_{ct}} \end{array}$$

Where *T* and *n* refer to the temperature and the electrons number, respectively. The exchange current density (*j*_0_) of LiFeBO_3_/C (10.78 × 10^−4^ mA cm^−2^) is higher than that of LiFeBO_3_ (7.26 × 10^−5^ mA cm^−2^), which indicates that LiFeBO_3_/C has a better electrode reaction reversibility than LiFeBO_3_.

**Table 2:** Parameters obtained from equivalent circuit fitting of EIS data.

**Sample**	***R*_**s**_(Ω)**	***R*_**ct**_(Ω)**	***j*_**o**_(mA cm^**−2**^)**
LiFeBO_3_	6.03	354.12	7.26 × 10^−5^
LiFeBO_3_/C	5.79	238.60	10.78 × 10^−5^

**Figure 6:**
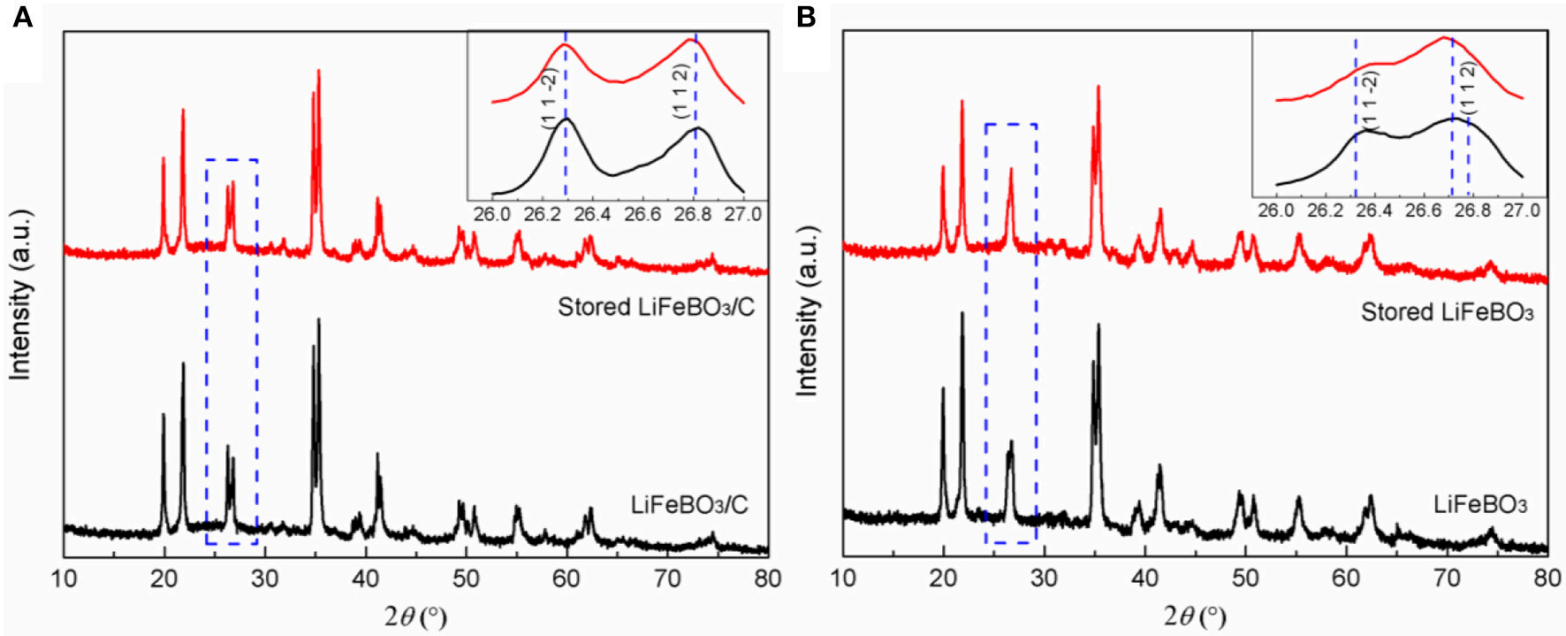
XRD patterns of LiFeBO_3_/C **(A)** and LiFeBO_3_ **(B)** before and after storing for 6 months (air exposure at room temperature).

To evaluate the storage stability, LiFeBO_3_/C and LiFeBO_3_ samples were stored in air exposure for 6 months. XRD patterns of the LiFeBO_3_/C and LiFeBO_3_ samples before and after storage are shown in Figure 6. Gratifyingly, the stored LiFeBO_3_/C sample also remains its original crystal structure, verifying high storage stability of LiFeBO_3_/C, as shown in Figure 6A. However, the XRD pattern of LiFeBO_3_ changed after storing 6 months. As can be seen from Figure 6B, the intensity of (1 1 2) peak tends to increase while the intensity of (1 1 2) peak tends to decrease, and the two peaks prone to overlap each other. This phenomenon can be attributed to phase changing of LiFeBO_3_ from monoclinic to orthorhombic structure with air corrosion (Bo et al., 2012; Chen et al., 2015).

**Figure 7:**
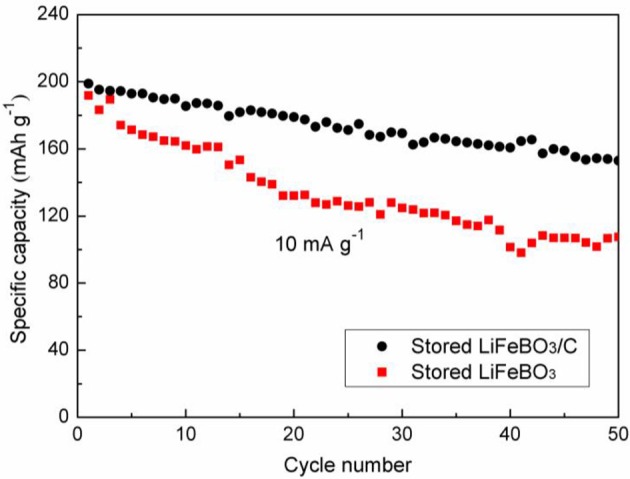
Cycling performances of the LiFeBO_3_/C and LiFeBO_3_ after storing for 6 months.

After 6 months of storage, the cycling performances of LiFeBO_3_/C and LiFeBO_3_ samples are investigated, and the results are shown in Figure 7. The capacity of the stored LiFeBO_3_/C is as high as 198.9 mAh g^−1^ and there is 76.9% capacity retention after 50 cycles. However, the stored LiFeBO_3_ delivers a capacity of 191.7 mAh g^−1^ and only 56.1% capacity retention after 50 cycles. Obviously, the stored LiFeBO_3_/C shows higher stability and cycling performance than the stored LiFeBO_3_. In summary, carbon coating and structural modification improve the conductivity of LiFeBO_3_, shorten the Li^+^ diffusion/conduction path and protect LiFeBO_3_ from moisture corrosion, thus leading to good electrochemical performance.

## Conclusions

LiFeBO_3_/C hollow sphere is successfully synthesized by a facile spray-drying method. Primary LiFeBO_3_@C particles are connected by carbon layers and formed LiFeBO_3_/C hollow spheres with improved electrical conductivity. Meanwhile, the hollow porous structure boosts the Li^+^ diffusion and the carbon layers of LiFeBO_3_@C particles protect LiFeBO_3_ from moisture corrosion. Therefore, the synthesized LiFeBO_3_/C sample shows good electrochemical performances and improved storage performance. This work is conducive to obtaining promising and high-performance cathode materials for LIBs.

## Data Availability

The raw data supporting the conclusions of this manuscript will be made available by the authors, without undue reservation, to any qualified researcher.

## Author Contributions

YS, ST, and WC did the main experiment and write the manuscript. LW and BW envolved the discussion of the experiment and revised the manuscript. HL and WL assisted the material synthesis. LW and SZ made the research plan. SZ and LW also provided the financial support.

### Conflict of Interest Statement

The authors declare that the research was conducted in the absence of any commercial or financial relationships that could be construed as a potential conflict of interest.
